# Performance enhancement of GaN-based light emitting diodes by transfer from sapphire to silicon substrate using double-transfer technique

**DOI:** 10.1186/1556-276X-7-244

**Published:** 2012-05-06

**Authors:** Jiang-Yong Zhang, Wen-Jie Liu, Ming Chen, Xiao-Long Hu, Xue-Qin Lv, Lei-Ying Ying, Bao-Ping Zhang

**Affiliations:** 1 Laboratory of Micro/Nano Optoelectronics, Department of Physics and Semiconductor Photonics Research Center, Xiamen University, Xiamen 361005, Fujian, People's Republic of China; 2 Pen-Tung Sah Micro/Nano Technology Research Center, Xiamen University, Xiamen 361005, Fujian, People's Republic of China

**Keywords:** GaN, Transferred light-emitting diodes, Thermal dissipation, Light extraction, Double-transfer, 81.05.Ea, 85.60.Bt, 85.60.Jb

## Abstract

GaN-based light emitting diodes (LEDs) fabricated on sapphire substrates were successfully transferred onto silicon substrates using a double-transfer technique. Compared with the conventional LEDs on sapphire, the transferred LEDs showed a significant improvement in the light extraction and thermal dissipation, which should be mainly attributed to the removal of sapphire and the good thermal conductivity of silicon substrate. Benefited from the optimized wafer bonding process, the transfer processes had a negligible influence on electrical characteristics of the transferred LEDs. Thus, the transferred LEDs showed a similar current–voltage characteristic with the conventional LEDs, which is of crucial importance for practical applications. It is believed that the double-transfer technique offers an alternative way to fabricate high performance GaN-based thin-film LEDs.

## Background

As one of the most important light source in next-generation solid-state lighting, GaN-based light emitting diodes (LEDs) have been extensively developed in past few years. Nowadays, GaN-based LEDs have already been used extensively in traffic signals, full-color displays, and backlight units for liquid crystal displays. To further extend the application arm of GaN-based LEDs to general lighting, further improvement on heat dissipation and light extraction efficiency are eagerly required. However, the poor thermal conductivity of sapphire prevents efficient dissipation of heat generated from the active area during operation, thus inducing seriously junction heating and the subsequent reduction of internal quantum efficiency. Moreover, the low light extraction efficiency caused by the absence of bottom reflector is also a problem associated with conventional GaN-based LEDs.

To overcome these problems, GaN-based thin-film LEDs with a bottom reflector fabricated by wafer bonding and laser lift-off (LLO) techniques have been proposed [1]. Significant improvements of light extraction and heat dissipation have been reported from the GaN-based thin-film LEDs [2]‐[6]. However, the fabrication procedures of such LEDs were complicated and would influence the performance of the devices, among which the increased reverse leakage current caused by the increase of screw dislocations is one of the most serious problems [6,7]. There have been several reports showing that the reverse leakage currents of thin-film LEDs were over three times greater than those of conventional LEDs [6]‐[8]. Although enormous efforts have been devoted to avoid electrical degradation in GaN-based thin-film LEDs, so far, the effective solution is still lacking. Previously, there has been intensive research into eliminating the negative influence of the LLO process [7]‐[9], but little work has been done to consider the effects of wafer bonding. Actually, this topic is of fundamental importance because the wafer bonding is a crucial process for thin-film LEDs which would have an important influence on electrical characteristics of the devices. To optimize the wafer bonding process may be an alternative way to solve the problem of electrical degradation in GaN-based thin-film LEDs. In this work, a double-transfer approach consisted of LLO and optimized wafer bonding processes was introduced to fabricate GaN-based thin-film LEDs. Low reverse leakage currents, high light extraction, and superior thermal dissipation were demonstrated in the fabricated devices.

## Methods

**Figure 1:**
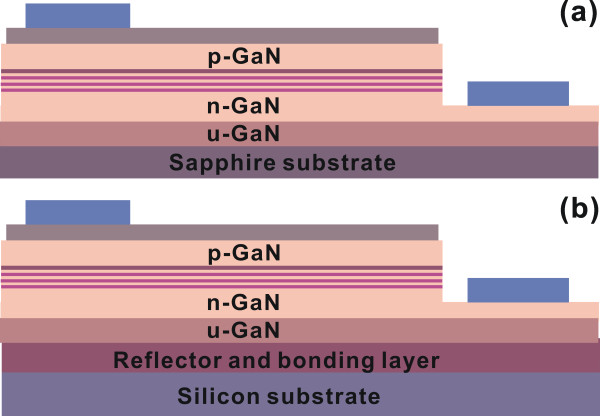
**Schematic of conventional LEDs and transferred LEDs.** (**a**) The conventional LEDs on sapphire substrate and (**b**) the transferred LEDs on silicon substrate.

The LED epilayers were grown on a 0001-oriented sapphire substrate by metalorganic chemical vapor deposition. The conventional LED chips were fabricated by a regular chip process with 300 × 300 μm^2^ mesa size. The finished chip structure is shown in Figure 1a. Next, a double-transfer approach was used to transfer the conventional LEDs onto a silicon substrate. The fabricated LEDs on sapphire substrate were first bonded to a temporary substrate using glue under 80°C for 5 h without imposed pressure. Subsequently, the sapphire substrate was removed by the LLO technique using a 248-nm KrF laser. Then, an Al-based reflective mirror was deposited on the exposed back side of the LEDs. Afterwards, the devices were bonded onto a silicon permanent substrate by means of Sn fusion-bonding technique at the temperature of 250°C and pressure of 0.5 MPa in vacuum for 10 min. Finally, a removal of temporary substrate and glue was performed, and the GaN-based thin-film LEDs with p-side up configuration were obtained. The final structure of transferred LEDs is shown in Figure 1b. It is worth mentioning that the double-transfer technique allows all the device fabrications to be made on the sapphire substrate right after growth, and no further device fabrication process was required after the transfer. This could significantly improve the process yield and reliability of the GaN-based thin-film LEDs.

Current–voltage (I-V) characteristics of the fabricated devices were measured using a semiconductor parameter analyzer. The light emitted from the top surface of the chip was collected by a silicon detector mounted directly above the devices. All the device characterizations were conducted on more than ten LED devices throughout the entire sample. It was observed that both the conventional LEDs and transferred LEDs had good uniformity over most area of the sample; thus, these data presented below are all representative results.

## Results and discussion

**Figure 2:**
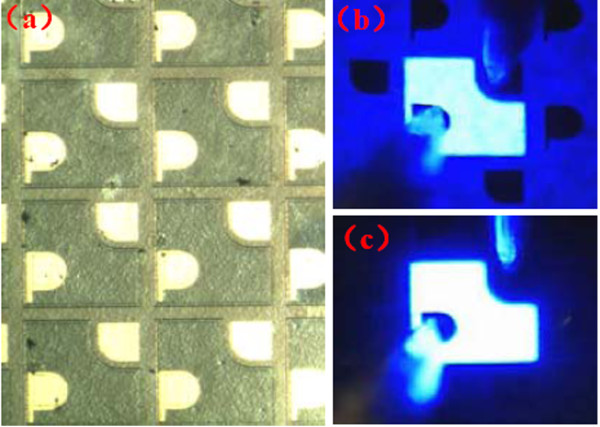
**Photomicrograph and emission images.** (**a**) Plain view photomicrograph of the transferred LEDs. Emission images of (**b**) the conventional LEDs and (**c**) the transferred LEDs at 1 mA.

Figure 2a shows the optical micrograph of the transferred LEDs on silicon substrate. A noted feature from this image is that, after double-transfer, the entire membrane was still very intact and without any signs of microcrack over the entire device area, indicating that the transfer process employed in this work introduces negligible extra stress. The light emission images of conventional LEDs and transferred LEDs at 1 mA are shown in Figure 2b,c, respectively. It can be seen that the light emission was distributed uniformly over the entire surface of each device even at a very low current. A comparison shows that the transferred LEDs have a higher concentrated brightness with no spreading of light, indicating an efficient enhancement of the light extraction efficiency.

**Figure 3:**
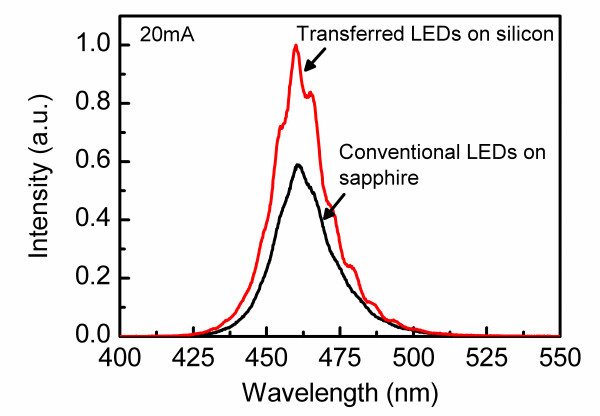
Typical room-temperature electroluminescence spectra of the conventional LEDs and the transferred LEDs at 20 mA.

Figure 3 shows the room-temperature electroluminescence spectra of conventional LEDs and transferred LEDs at 20 mA. It can be seen that the emission spectrum of transferred LEDs shows clear interference fringes, and the light emission intensity is obviously greater than that of conventional LEDs. This enhancement in light intensity at relatively low-level injection current is mainly due to the effective reflection of bottom reflector.

**Figure 4:**
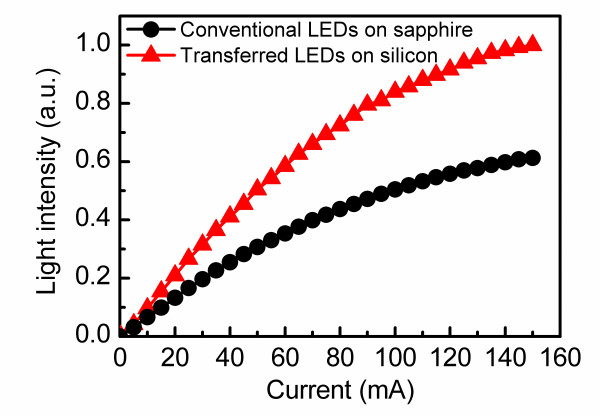
Light intensity versus injection current characteristics of the conventional LEDs and the transferred LEDs.

Figure 4 shows the light intensity versus injection current characteristics of the conventional LEDs and the transferred LEDs. It can be found that the transferred LEDs showed distinct enhancement in the light intensity under all our measurement conditions. Compared with that of conventional LEDs, the light intensity of transferred LEDs increases by 58 % at 20 mA. However, when the injection current increased to 150 mA, the enhancement in light intensity can reach 63 %. This result means that the light intensity of transferred LEDs increases greater than that of conventional LEDs with increasing current, which should be mainly attributed to the superior thermal dissipation capability of the transferred LEDs.

**Figure 5:**
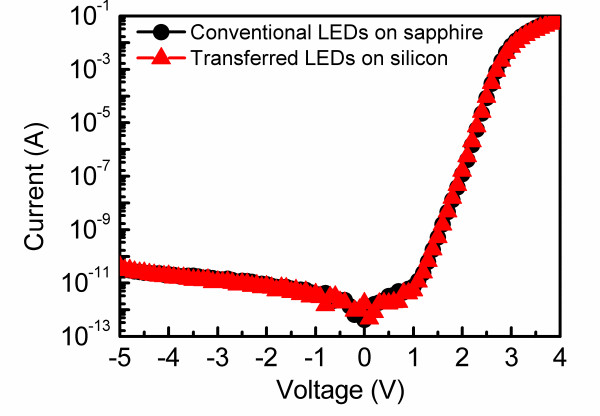
Current–voltage characteristics of the conventional LEDs and the transferred LEDs.

Figure 5 shows the typical I-V characteristics of the conventional LEDs and the transferred LEDs. It can be seen that both devices have similar I-V characteristics. At a driving current of 20 mA, the forward voltages of conventional LEDs and transferred LEDs were 3.25 and 3.31 V, respectively. The almost identical forward voltages are ascribed to the same epitaxial layers and electrode configuration used in these two devices. More importantly, in the reverse biased region, the leakage currents of the conventional LEDs and transferred LEDs are almost identical. These results confirm that the electrical property of the devices were not degraded during the double-transfer processes described earlier, which is crucial of importance for practical applications.

The optical and electrical measurements of the transferred LEDs have revealed that the electrical degradation in thin-film LEDs can be avoided by utilizing the wafer bonding process proposed here. This considerable improvement should be mainly attributed to the effective suppression of residual stress at bonding interface using the low temperature and pressure-free glue bonding. It is well known that the LED epilayers and bonding metal always have a high thermal mismatch because of their difference in thermal expansion coefficient. As a result, the high temperature and pressure procedures during mental bonding would lead to a large residual stress at the bonding interface. After the sapphire removal, the thin LED epilayers are very brittle and favorable to generation of defects, thereby, increasing reverse leakage current. Moreover, the thickness of p-GaN cladding top layer is considerably thin, only about 0.4 μm. The requisite treatment for mental bonding would result in damage to the MQWs active region and the consequent electrical deterioration. Therefore, the glue bonding with low temperature and pressure-free processes is highly beneficial to avoid these negative influences. In addition, the relative lower temperature and pressure during Sn fusion bonding, the thick n-GaN and u-GaN layers, and the bottom mental reflector could effectively prevent damage of the MQWs active region during the second wafer bonding after LLO, which also play important roles in eliminating the electrical degradation of thin-film LEDs.

## Conclusions

In summary, GaN-based LEDs fabricated on sapphire substrates were transferred onto silicon substrates using a double-transfer technique. The transferred LEDs exhibited a significant improvement in the light extraction and thermal dissipation as compared with that of conventional LEDs on sapphire. This improved performance was ascribed to the removal of sapphire and the good thermal conductivity of silicon. The electrical measurement demonstrated that the transferred LEDs have negligible changes in forward voltage and reverse leakage current, indicating that the fabrication processes would not deteriorate the electrical property of the devices. Considering the fabrication processes of the devices, the effective suppression of electrical degradation in transferred LEDs should be mainly attributed to the improvement of wafer bonding process. It is believed that the double-transfer technique offers an alternative way to fabricate high performance GaN-based thin-film LEDs.

## Abbreviations

LEDs = Light emitting diodes; LLO = Laser lift-off; I-V = Current–voltage.

## Competing interests

The authors declare that they have no competing interests.

## Authors' contributions

The work presented here was carried out in collaboration among all authors. JYZ and BPZ designed the study. JYZ performed the research and prepared the manuscript. WJL, MC, XLH, XQL, and LYY analyzed the data and discussed the analysis. All authors read and approved the final manuscript.
